# Manual cervical therapy and vestibular migraine: A case series

**DOI:** 10.12688/healthopenres.13319.1

**Published:** 2023-05-22

**Authors:** Roger O'Toole, Dean Watson

**Affiliations:** 1 Melbourne Headache Centre, Melbourne, Victoria, 3000, Australia; 2 Allied Health and Human Performance, University of South Australia, Adelaide, South Australia, Australia

**Keywords:** Migraine, dizziness, vertigo, upper cervical spine, manual pressure, musculoskeletal dysfunction, physiotherapy, neck treatment, complementary and integrative medicine.

## Abstract

**Background:**

Vestibular migraine (VM) is a relatively new diagnostic entity with incomplete knowledge regarding pathophysiological mechanisms and therapeutic guidelines. By reporting the effect of manual cervical therapy (MCT) on people with VM, we suggest a possible role for upper cervical afferents in VM treatment and/or pathogenesis. The objective was to describe the change in clinical presentation and self-reported symptoms of VM corresponding to MCT and followed up to six months.

**Methods:**

A nonrandomised *ABA design was utilised to consecutively and prospectively evaluate selected patients with diagnosed VM. Symptom characteristics (frequency and intensity) were recorded along with standardised patient-reported outcomes (PROs) to document the response to MCT.

**Results:**

Three patients were recruited who met the diagnostic criteria for VM. All three patients demonstrated improvement in both migraine attack and interictal symptom frequency. These improvements mirrored changes in PROs and were sustained over a six-month follow-up period.

**Conclusions:**

The improvement that coincided with the intervention including MCT was rapid, observable and sustained. This suggests that the upper cervical spine could be a therapeutic target in VM and may have implications for future research into the pathogenesis of VM.

## Plain language summary

Migraine is a complex disorder with many different factors potentially contributing to a person developing the condition. Whilst many different parts of the brain and body have been implicated as potential causes, the neck is often overlooked. To provide the best possible solution to patients suffering all forms of migraine, including vestibular migraine, a broader discussion must include all potential factors that may contribute to the disease developing, and/or contribute to optimal management. This article provides support for the upper neck to be included in that conversation, and provides the basis for further research to determine the significance of the neck in causing and/or treating this disorder.

## Introduction

Vestibular migraine (VM) is a disorder characterised by episodes of specific vestibular symptoms, at least half of which are accompanied by features consistent with migraine ( Lempert *et al.*, 2012), and is considered to be the most frequent cause of episodic vertigo ( Dieterich *et al.*, 2016).

The diagnostic criteria for VM were formalised jointly by the Committee for Classification of Vestibular Disorders of the Bárány Society and the International Headache Society in 2012 ( Lempert *et al.*, 2012); hence, as a relatively new diagnostic entity, information regarding epidemiology is limited. The one-year prevalence of VM (including ‘probable’ VM) is estimated to be between 1.98% ( Neuhauser *et al.*, 2006) and 2.7% ( Formeister *et al.*, 2018); however, this underestimates the relationship between migraine and vestibular symptoms. Comorbidity of migraine in patients attending dizziness clinics has been reported to be as high as 38% ( Neuhauser *et al.*, 2001). As many as 58.9% of patients attending migraine clinics report moderate to severe vestibular symptoms ( Kayan & Hood, 1984), which increases to 72.4% when mild symptoms are considered ( Kelman & Tanis, 2006).

The current hypotheses of the pathophysiology of VM relate to abnormal thalamic function, calcitonin gene-related peptide release into the inner ear and/or brainstem and/or cerebellum, genetic factors, abnormal processing of shared vestibular and nociceptive pathways, and cortical spreading depression ( Furman *et al.*, 2013). The role of the neck in cervicogenic dizziness is well established ( Reiley *et al.*, 2017), with some phenotypic expressions of whiplash-associated disorder bearing striking similarity to VM. Patients frequently report symptoms of dizziness and/or unsteadiness (90%), headache (56%), nausea (40%) and spinning (25%) despite a lack of positive otoneurological findings ( Treleaven *et al.*, 2003). Case reports of cervicogenic vertigo ( Brandt & Huppert, 2016) remain contingent on a temporal relationship to neck pain; however, experimental findings support an association between nonpainful upper cervical afferents and vestibular symptoms. Blocking afferents by injecting anaesthetic unilaterally in the upper cervical spine induced vertigo (sense of titling and falling) ipsilaterally ( de Jong *et al.*, 1977), whilst mimicking activation of cervical muscle spindles with vibratory stimulation results in both the visual environment moving contralaterally to the vibration ( Jamal *et al.*, 2020), and the sense of self-motion being either doubled (activated ipsilateral to side of rotation) or annulled (activated contralateral to side of rotation) ( Panichi *et al.*, 2011).

Patients with migraine show greater musculoskeletal dysfunction than healthy controls ( Luedtke *et al.*, 2018), including abnormal electromyography (EMG) findings related to the upper cervical musculature ( Bakal & Kaganov, 1977; Janani *et al.*, 2018). The impact of upper cervical muscle dysfunction on the vestibulo-visual system is underscored by their exceedingly high muscle spindle density ( Kulkarni *et al.*, 2001) and their convergence and antagonism with vestibular afferents at all levels of the central nervous system (CNS), including the central cervical nucleus ( Hongo *et al.*, 1988), external cuneate nucleus ( Anastasopoulos *et al.*, 1991), vestibular nuclei ( Fredrickson *et al.*, 1966), superior colliculus ( Maeda *et al.*, 1979), thalamus ( Liedgren *et al.*, 1976), cerebellum ( Berthoz & Llinás, 1974) and parieto-insular vestibular cortex ( Shinder & Newlands, 2014).

The widely accepted model describing neural integration of afferent inflow from the vestibular, visual and proprioceptive systems was first proposed by von Holst and Mittelstaedt ( von Holst & Mittelstaedt, 1950), whereby an ‘efference copy’ of the intended movement is subtracted from the feedback from self-generated movement or ‘reafference’. A perfect match results in sensory inflow from the vestibular system being cancelled ( Figure 1a). Brooks and Cullen ( Brooks & Cullen, 2014) demonstrated that aberrant feedback from upper cervical muscles failed to cancel vestibular reafference, leading to a summation of vestibular signals ( Figure 1b) and resulting in vestibular symptoms.

**Figure 1a.**
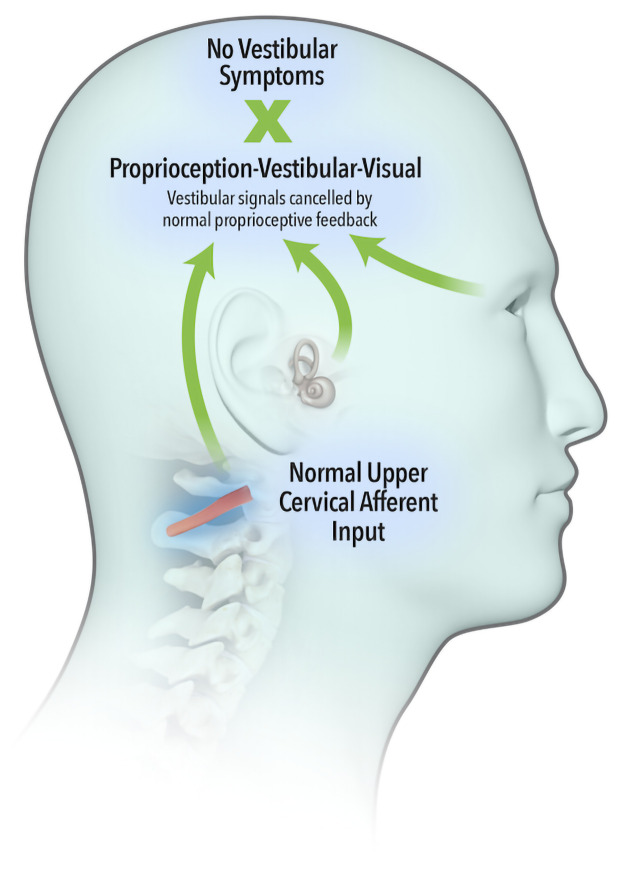
Sensory inflow from the vestibular system cancelled by normal and expected proprioceptive inputs.

**Figure 1b.**
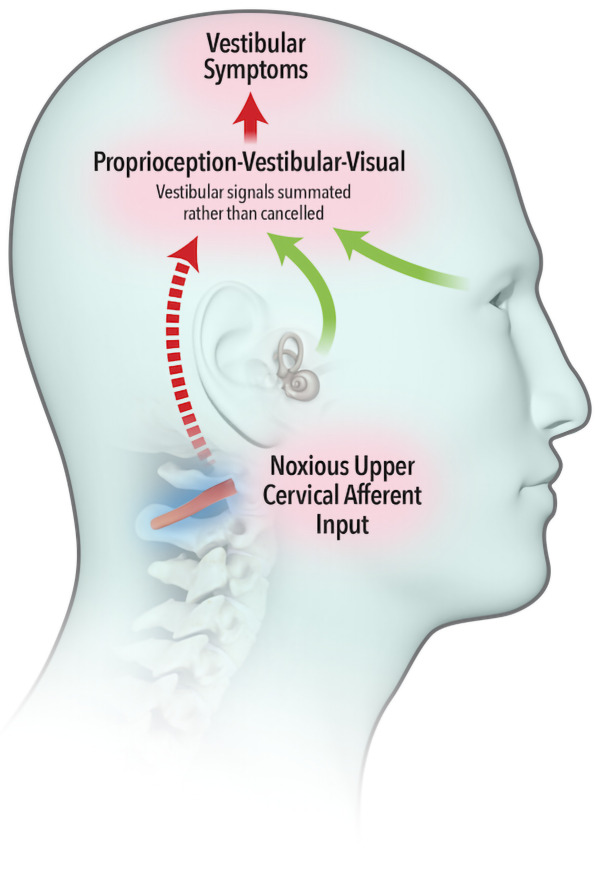
Noxious afferent feedback from upper cervical muscles fails to cancel sensory inflow from the vestibular system. Vestibular symptoms are experienced.

Additional upper cervical afferents project to CNS regions believed to be involved in the pathophysiology of migraine. These include the trigeminocervical complex ( Piovesan *et al.*, 2003), hypothalamus ( Malick *et al.*, 2000), locus coeruleus ( Cedarbaum & Aghajanian, 1978) and vestibular nuclei ( Fredrickson *et al.*, 1966) along with centres involved in autonomic regulation (nucleus tractus solitarius ( Menétrey & Basbaum, 1987)), providing a compelling anatomical and physiological case for upper cervical afferent contribution to the symptomatic manifestation of VM.

Given the lack of double-blinded, randomised placebo-controlled trials for acute or prophylactic medication use in VM, it is understandable that current approaches to treatment are largely based on the therapy guidelines for migraine without aura ( Byun *et al.*, 2021). Nonpharmacological interventions, including neurostimulation, counselling, relaxation techniques, biofeedback, and physical therapy vestibular rehabilitation (VRT), have been suggested ( Obermann & Strupp, 2014), with VRT showing small but significant improvements ( Alghadir & Anwer, 2018). Despite attempts to identify ‘gaps’ in knowledge ( Mallampalli *et al.*, 2021) and the body of research linking upper cervical afferents to the production of vestibular symptoms in clinical, experimental and anatomical studies, their potential contribution to the pathophysiology of VM is consistently omitted, and MCT is not currently considered a therapeutic option.

The aim of this case series is to demonstrate the effect of MCT on the clinical presentation and self-reported symptoms of VM and thus provide preliminary evidence in support of MCT as a novel and plausible modality for the treatment of VM and a basis for further research examining the role of upper cervical afferents in VM pathophysiology.

## Methods

This study protocol was developed in line with the CARE guidelines for reporting on case reports ( Gagnier *et al.*, 2013) and the CENT guidelines for reporting on N-of-1 trials ( Vohra *et al.*, 2015) in an attempt to minimise bias. Ethics approval was granted by the University of South Australia Human Research Ethics Committee (protocol number: 202637).

### Trial design

A case-series methodology ( Vohra *et al.*, 2015) was employed with a nonrandomised *ABA design, mimicking that of an N-of-1 study. Period ‘*A’ (i.e., run-in) was ‘usual care’ and varied in length based on the clinic waiting list and participant availability to commence treatment. The duration of period *A and the nature of ‘usual care’ were not standardised for ethical reasons, as this would have involved delaying treatment. Period ‘B’ was MCT. As is standard in physiotherapy practice, the nature, frequency and timing of the interventions were not standardised and were based on participant availability and treatment response in concert with an individual’s unique VM presentation. Period ‘A’ was postintervention. Participants did not commence any new interventions or change medications that could influence their VM symptoms throughout the data collection period (*ABA).

### Eligibility criteria

Three participants (two females aged 26 and 48, a male aged 43) who were diagnosed with VM by a neurologist and met the Bárány Society criteria for VM ( Lempert *et al.*, 2012) sought treatment from the participating physiotherapist (RO). Participants were excluded from the study if they had a history of previous neck surgery or traumatic head injury, recently started or changed medication or other treatment with the potential to influence VM (i.e., antidepressant medication, anxiolytics, analgesics, vestibular suppressants, VRT), had recently started or changed MCT to the cervical spine, or were diagnosed with another chronic pain condition (i.e., fibromyalgia, complex regional pain syndrome). Any potential participants who were unable to read the information sheet or demonstrate understanding of the study information presented if read to them or had insufficient cognitive capacity to provide voluntary written informed consent or impaired capacity to communicate verbally (i.e., aphasia) were excluded.

### Study setting and recruitment

The study took place at a private physiotherapy clinic in Australia. Participants were prospectively identified at the time of booking their initial consultation. Administration or clinical staff screened each patient against the Bárány Society criteria for VM ( Lempert *et al.*, 2012) and the study criteria. Subsequently, absence of neck pain and/or limitation of cervical ROM in all three participants eliminated concurrent ‘cervicogenic dizziness’ as an alternate or comorbid diagnosis ( Reiley *et al.*, 2017). All potential participants were treated as study recruits; however, to avoid perceived coercion, individuals were not formally invited to participate until the end of period B. To avoid reporting bias, after period B, all prospectively identified individuals were invited to participate regardless of treatment response.

### Outcomes and analysis

The primary outcome measures were symptom severity and frequency, and the use of pharmaceutical intervention, recorded by a patient diary in periods A* and A, and therapist notes in period B. A 50% or greater change in symptom frequency over a 28-day period was considered significant. Patient-reported outcomes (PROs) were used as secondary outcome measures and included the Headache Impact Test-6 (HIT-6), Dizziness Handicap Inventory (DHI), and the Depression Anxiety Stress Scale (DASS-21).

The impact of headache on daily life was assessed using the HIT-6, which is a validated six-item questionnaire generating total scores from 36 to 78, with scores ≥ 60 indicating severe impact (for full scoring see ( Yang *et al.*, 2011)). In people with migraine, the within-person clinically meaningful change in the HIT-6 score over 12 weeks is estimated to be between -2.5 (mean change approach) and -6 points (ROC curve approach) ( Smelt *et al.*, 2014). We have used the more conservative six-point change to indicate improvement.

The DHI ( Jacobson & Newman, 1990) is a validated 25-item scale developed to assess the handicap caused by dizziness, with total scores ranging from 0 to 100. Total scores are divided into three handicap categories from mild (0-30) to moderate (31-60) and severe (61-100) ( Whitney *et al.*, 2004). There is a standard error for test-retest indicating a minimal important change of 18 points from pre- to postintervention and three points using the ROC curve approach ( Friscia *et al.*, 2014).

The DASS-21 is a valid and reliable measure used to detect symptoms of depression, anxiety and stress ( Antony *et al.*, 1998), with subscales having independent cut-off scores (reported in ( Lovibond & Lovibond, 1995)) for symptom severity that can be averaged to produce a composite measure. Minimum detectable change has been reported for each subscale as 3.86 (depression), 3.85 (anxiety) and 4.9 (stress) ( Ronk *et al.*, 2013).

Clinical records were audited at the conclusion of each participant’s intervention period. Primary and secondary outcome measure data were extracted and input into Excel© (Microsoft, USA) along with details of the intervention delivered. Changes in VM attack features (frequency, severity and medication use) and impact (HIT-6, DHI and DASS-21) are presented descriptively as a series of cases using measures of ‘clinically meaningful change’ ( Jacobson & Newman, 1990; Ronk *et al.*, 2013; Smelt *et al.*, 2014).

### Interventions

No manipulative (localised, high-velocity thrust, low amplitude) techniques were applied. During period B, participants received an MCT intervention from a physiotherapist registered in Australia. In accordance with a specific protocol based on techniques described in part by Watson and Drummond ( Watson & Drummond, 2014), the therapist applied sustained digital ‘postero-anterior’ or PA pressure to the C3 spinous process ( Watson, 1997). The direction and depth of pressure and the number and duration of holds were unique to each individual and appropriate for the participants’ presentations (determined by manual assessment of the neck).

Due to the nature of a case series, data analysis was not conducted. However, to provide a description of change in self-reported measures pre- and postintervention, we used Hedges g effect size analysis ( Shadish *et al.*, 2015) with the magnitude of change interpreted using Cohen’s d recommendations of trivial (<0.2), small (0.2-0.49), medium (0.5-0.79) and large (>0.8) ( Cohen, 1988). All collated data and effect size calculations are provided in Supplementary file 1.

## Results

### Cases and eligibility

Initially, four individuals were enrolled. However, one individual was excluded from the study prior to assessment due to the presence of comorbid fibromyalgia. Three eligible individuals reported having had at least five attacks, and all reported headache and visual and vestibular symptoms as features of their migraine presentation (see Table 1, Table 2, Table 3). All participants tolerated the intervention, and no contraindications or adverse events related to the intervention were reported.

**Table 1.** Participant 1 Symptom features pre- and postMCT.

TIMEPOINT (W = week)	*W _0_* * *A - Run-in*	*W _5_* *B - Treatment* * (Rx) block*	*W _9_* *A - 1 month* * post-Rx*	*W _17_* *3 months* * post-Rx*	*W _29_* *6 months* * post-Rx*
***Average*** *** intensity (NRS)*** ***Headache***	5/10	3/10	2/10	2/10	4/10
	***Attack/symptom frequency per 28 days***				
	***28 day diary***	First 28 days of Rx block	1st 28 days post-Rx	28 day diary 2-3 months post-Rx	28 day diary 5-6 months post-Rx
***Medication***	Effexor 30 mg Propranolol 80 mg	Effexor 30 mg Propranolol 80 mg	Effexor 30 - 35 mg Propranolol 100 mg	Effexor 35 - 37.5 mg Propranolol 120 -160 mg	Effexor 37.5 mg Propranolol 160 mg
***Migraine***	6	1 *	0	0	0
***Headache***	20	10 *	6	1	11
***Nausea***	8	7	4 *	1	3
***Dizziness***	25	5 *	10	6	3
***Internal Vertigo***	4	2 *	1	0	0
***External Vertigo***	15	4 *	0	0	1
***No Symptoms***	0	12 *	14	21	11
***HIT-6***	67	55 *	46	61 *	49 *
***DHI***	76	44 *	24	40 *	26 *
***DASS-21***	D-22 ^(S)^, A-24 ^(ES)^, S-32 ^(S)^	D-6 ^(N)*^ A-6 ^(N)*^ S-10 ^(N)*^	D-0 ^(N)*^ A-2 ^(N)*^ S-4 ^(N)*^	D-14 ^(Mo)*^ A-8 ^(Mi)*^ S-14 ^(N)*^	D-2 ^(N)*^ A-4 ^(N)*^ S-12 ^(N)*^

Rx (Treatment) I NRS (Numerical Rating Scale for pain) I *clinically meaningful change | (ES) Extremely severe, (S) - severe, (Mo) moderate, (Mi) mild, (N) normal

**Table 2.** Participant 2 Symptom features pre- and postMCT.

TIMEPOINT (W = week)	*W _0_* *A - Run-in period	*W _4_* *B - Rx block*	*W _12_* *A - 1 month * *post-Rx*	*W _23_* *3 months* * post-Rx*	*W _39_* *6 months* * post-Rx*
***Average intensity (NRS)*** ***Headache*** ***Dizziness*** ***Nausea***	5/10 4/10 5/10	5/10 3/10 3-5/10	1-2/10 0-1/10 0-1/10	3-4/10 3-4/10 3-5/10	1-2/10 0-1/10 4-5/10
	***Attack/symptom frequency per 28 days***				
	***25 day diary*** ***(equivalent/28*** *** days)***	First 28 days of Rx block	28 day diary post-Rx	28 day diary 2-3 months post-Rx	28 day diary 5-6 months post-Rx
***Medication***	Nil	Nil	Nil	1 x metoclopramide	Nil
***Migraine***	14 (16/28)	1 *	0	0	0
***Headache***	15 (17/28)	10	3 *	5	2
***Nausea***	19 (21/28)	11	3 *	7	7
***Dizziness***	18 (20/28)	10 *	3	5	3
***Fatigue***	7 (8/28)	10	3 *	5	3
***Symptom free days***	4 (4/28)	16 *	25	16	17
***HIT-6***	63	48 *	36	52 *	36
***DHI***	72	28 *	14	28	26
***DASS-21***	D-0 ^(N)^ A-8 ^(Mi)^ S-16 ^(Mi)^	D-2 ^(N)^ A-4 ^(N)*^ S-10 ^(N)*^	D-0 ^(N)^ A-2 ^(N)^ S-8 ^(N)^	D-4 ^(N)^ A-2 ^(N)^ S- 8 ^(N)^	D- 0 ^(N)^ A- 0 ^(N)^ S- 8 ^(N)^

Rx (Treatment) I NRS (Numerical Rating Scale for pain) I *clinically meaningful change | (ES) Extremely severe, (S) - severe, (Mo) moderate, (Mi) mild, (N) normal

**Table 3.** Participant 3 Symptom features pre- and postMCT.

TIMEPOINT (W = week)	*W _0_* *A - Run-in	*W _9_* *B - Rx block*	*W _11_* *A - 1 month* * post Rx*	*W _21_* *3 months * *post Rx*	*W _33_* *6 months * *post Rx*
***Average intensity (NRS)*** ***Headache*** ***Dizziness***	3/10 4-5/10	3/10 3/10	2/10 0/10	2/10 1-2/10	5/10 4/10
	***Attack/symptom frequency per 28 days***				
	***16 day diary*** ***(equivalent /28*** *** days)***	First 28 days of Rx block	First 28 days post Rx	28 day diary 2-3 months post Rx	28 day diary 5-6 months post Rx
***Medication***	Daily (28/28) Ibuprofen 500mg Paracetamol 1000mg Circadin 2mg	Ibuprofen 9 * Panadol 10 *	Ibuprofen 3 * Panadol 0 *	Ibuprofen - 10	Ibuprofen 0 * Maxigesic x 3 Maxalt x 1
***Migraine***	4 (7/28)	0 *	0	0	0
***Headache***	13 (23/28)	15	3 *	0	3
***Dizziness***	13 (23/28)	20	0 *	10	2
***Internal Vertigo***	4 (7/28)	0 *	0	0	0
***External Vertigo***	15 (26/28)	3 *	1	0	3
***Symptom free days***	0	4 *	25 *	18	25
***HIT-6***	73	74	56 *	57	59
***DHI***	80	80	20 *	42	34
***DASS-21***	D-34 ^(S)^ A-20 ^(ES)^ S-22 ^(Mo)^	D-40 ^(ES)^ A-16 ^(S)^ S-18 ^(Mi)^	D-10 ^(Mi)*^ A-2 ^(N)*^ S-2 ^(N)*^	D-10 ^(Mi)*^ A-4 ^(N)*^ S-2 ^(N)*^	D- 0 ^(N)*^ A-0 ^(N)*^ S-1 ^(N)*^

Rx (Treatment) I NRS (Numerical Rating Scale for pain) I *clinically meaningful change | (ES) Extremely severe, (S) - severe, (Mo) moderate, (Mi) mild, (N) normal

### Participant 01 (P1)

***Participant profile.*** P1 was a 26-year-old female with a three-year history of vestibular symptoms after contracting a viral illness while recovering from surgery. Initially, diagnosed with vestibular neuritis, P1 experienced frequent headache, suboccipital tension (without neck pain), orthostatic and head motion-induced dizziness, spontaneous vertigo, ‘slow, unclear and fuzzy’ vision, and nausea. Subsequently, diagnosed with anxiety, treatment with a psychologist was unsuccessful. Venlafaxine was prescribed after a neurologist diagnosed VM, resulting in a decrease in symptoms (reported as 70-80%), but P1 was still prone to acute exacerbations with triggers.

Venlafaxine was weaned after two years with symptoms stable for six to eight weeks before returning with daily interictal symptoms. Migraine prophylaxis with topiramate (not tolerated well and ceased after two weeks) and propranolol (80 mg) was recommenced seven months prior to assessment (start of period ‘B’) without improvement. Venlafaxine was reintroduced four months before assessment, with slight improvement in dizziness intensity but otherwise no obvious improvement in other symptoms.

Migraine attacks began with paraesthesia in both feet, progressing proximally through the legs and body, subsequently resolving to be replaced by vertigo, headache (NRS 8/10) and nausea. Attacks lasted from one to three days, easing to milder chronic daily headache (NRS 5/10) with vestibular symptoms, including spontaneous and orthostatic dizziness and sound-induced vertigo, every second day (see Table 1).

Key findings of manual assessment: Palpation of the spinous process of C2 demonstrated deviation to the left of the midline. Transverse pressure on the C2 spinous process revealed increased resistance from left to right when compared with right to left.

***Intervention.*** Manual cervical therapy as described in Methods was provided. P1 had seven treatments over five weeks starting in September 2020, with two three-month nontreatment periods concluding in April 2021. A reassessment was conducted at the end of each nontreatment period.

***Outcomes.*** Raw data are presented in Table 1, indicating improvement across all primary and secondary outcome measures. P1 reported improvement in attack frequency from six migraine days per month (28 days) to no migraine days in the six-month nontreatment (follow-up) period. Interictal symptoms also showed improvement, with no symptom-free days in the run-in period changing to 21 symptom-free days (every 28 days) during the follow-up period.

Secondary outcome measures all showed clinically meaningful improvements during the treatment phase and continued to improve after the first four weeks of the nontreatment phase. After reporting increased feelings of depression with significant life events during the nontreatment period, all scores regressed slightly for a short time before showing sustained improvement at the six-month follow-up.

### Participant 02 (P2)

***Participant profile.*** P2 was a 48-year-old female with a 14-year history of recurrent vertigo and head motion-induced dizziness triggering migraine attacks following surgery on a pituitary cyst. A bout of persistent motion sickness at age 14 was closely followed by a fall from a horse, resulting in low back pain (no head or neck trauma was reported). Recurrent headache and nausea developed twelve months later, progressively worsening over a three-year period, which included the onset of menstrual-related migraine without aura (age16 years). At this time, nausea was constant, and multiple diagnoses from reflux to anorexia were given after losing 30 kg in weight over a four-year period.

Migraine attacks increased intensity and frequency after the birth of her last child, and after surgery on a pituitary cyst 14 years ago, symptoms worsened to include a ‘hemiplegic’ aura (weakness and numbness affecting one upper limb, facial drooping and dysarthria) and vertigo. Soon thereafter, P2 spent a week of 15 hours per day studying, after which she experienced ‘atypical trigeminal neuralgia’. The new ‘normal’ was chronic daily headache with nausea and lethargy, with bouts of dizziness and disequilibrium preceding intense nausea and severe headache.

P2 was diagnosed with VM by a neuro-otologist in July 2019 and prescribed amitriptyline, which was not tolerated well. P2 preferred meditation and manual therapy (chiropractic) for the daily headaches and low back pain, which would provide a reduction of symptoms for one to two days at best.

Migraine attacks began with intensification of interictal symptoms (nausea, dizziness and lethargy) and infrequently a scintillating scotoma. The scotoma gave way to suboccipital tightness and intense pressure in the retro-orbital regions and paroxysmal sharp ‘ice pick’ pain around the right orbit. Acute attacks lasted between three and five days, easing to interictal symptoms of chronic daily headache (NRS 5/10), nausea (5/10), dizziness and/or vertigo (4/10) and lethargy (6/10).

Key findings of manual assessment: Palpation of the spinous process of C2 demonstrated deviation to the right of the midline. Transverse pressure on the C2 spinous process revealed increased resistance from right to left when compared to left to right. Sustained left cervical rotation resulted in increased anxiety before returning to neutral, which was followed by a mild feeling of the body being pulled to the right. In addition, standing balance, tandem stance with eyes closed (TS, EC) was unable to be maintained for > 10 seconds.

***Intervention.*** Manual treatment as described in Methods was provided. P2 had seven treatments starting in September 2020. The first five appointments were in the first two weeks, with the remaining two appointments over a two- and four-week period. A symptom diary and outcome measures (PRO) were recorded after successive three-month nontreatment periods concluding in March 2021.

***Outcomes.*** Raw data are presented in Table 2, indicating improvement across all primary and secondary outcome measures. P2 reported an immediate improvement during the assessment with standing balance (TS, EC) stable for 25 seconds and no feeling of anxiety or vertigo with return from sustained left rotation. Migraine attack frequency improved from 16 migraine days per month (per 28 days) to one migraine day during the treatment period and no migraine days in the eight months to the end of data collection. This is despite being admitted to the hospital with colitis during the first three-month nontreatment period, which is reflected in some of the outcome measures worsening during this period. Interictal symptoms also showed improvement with four symptom-free days in the run-in period, with three of these occurring immediately after two chiropractic appointments to an average of 17 symptom-free days in the last month of each treatment-free period.

Secondary outcome measures showed clinically meaningful improvements during the treatment phase and continued to improve after the first four weeks of the follow-up phase. An episode of colitis caused an increase in nausea frequency, with a minimal increase in dizziness episodes; however, the perceived disability from dizziness worsened slightly.

### Participant 03 (P3)

***Participant profile.*** P3 was a 43-year-old male with a twenty-month history of VM that began insidiously following the commencement of a new job, disrupted sleep with a newborn and a bout of gastrointestinal symptoms. Around the same time, he struck the back of his head on a tree branch while gardening. While there was no neck pain or restriction of movement, P3 suffered persistent dizziness, accompanied by mild bilateral tinnitus, visual lag and nausea. Over several weeks the nausea and tinnitus eased completely and gave way to a persistent headache and a slight blurring of vision and visual lag with scanning, all of which were rarely absent. Rolling in bed, looking down at his phone, pushing his daughter in a pram or swing would induce vertigo and migraine attacks. These were preceded by paraesthesia in the suboccipital and occipital regions, followed by intensification of the baseline bilateral pressure headache (NRS 3/10) to moderate or severe headache (NRS 8/10), accompanied by internal vertigo (sense of falling), phonophobia (inducing severe nausea and exacerbating vertigo), and photophobia. Attacks would last for the remainder of the day, only relieved by sleep, and were moderate to severe intensity, preventing him from taking his daughter out in the pram and completing household tasks. The participant admitted being anxious about triggering attacks and feeling depressed about the impact it was having on his ability to engage with his daughter and maintain fitness.

After being diagnosed by an ENT specialist with persistent postural perceptual dizziness, P3 was referred to a neurologist who diagnosed VM. Amitriptyline, pizotifen, topiramate and propranolol were unsuccessful, and circadin helped sleep quality but did not improve the other symptoms.

Key findings of manual assessment: Full pain-free cervical ROM. Palpation of the spinous process of C2 demonstrated deviation to the right of the midline. Transverse pressure on the C2 spinous process revealed increased resistance from right to left when compared to left to right. In addition, ‘visual lag’ occurred when returning to neutral after sustained right cervical rotation.

***Intervention.*** Manual treatment as described in Methods was provided. P3 was a fly-in fly-out oil rig engineer, and as such treatments were grouped initially according to his availability. After his initial consultation in September 2020 the next four treatments occurred during October before a two-week, and then a four-week review concluded in December 2020, with the first seven appointments occurring in the first five weeks. Symptom diary and outcome measures (PROs) were collected four weeks after the end of the last treatment and then again after two successive three-month nontreatment periods.

***Outcomes.*** Raw data are presented in Table 3, indicating improvement across all primary and secondary outcome measures. P3 reported improvement in attack frequency from two migraine days per month to no migraine days after treatment began, including during a six-month follow-up phase. Interictal symptoms also showed improvement, with P3 reporting no symptom-free days in the run-in period and averaging 18 days per month symptom-free in the last month of each three-month follow-up phase, which included 10 dizziness days after P3 had fallen off a roof. This was reinforced by a reduction in medication use, with only one maxalt taken as a preventive ‘insurance’ before an important interview, rather than due to symptoms/migraine attack.

Secondary outcome measures showed no change during treatment. During the first four weeks of the follow-up phase, secondary outcome measures improved dramatically; however, they showed some regression with the mild increase in dizziness after a fall from a ladder, reflected in the outcome measures at three months.

## Discussion

The results of this case series demonstrate medium to large effects (Hedges’ g) of MCT on the clinical features (frequency, intensity, medication use) and self-reported symptoms (HIT-6, DHI, DASS-21) of VM in three consecutive and prospectively selected participants (see Supplementary file 1). Furthermore, no adverse reactions to treatment were reported. Reductions in all symptoms (migraine attacks and interictal symptoms) as well as PROs maintained medium to large effects over successive three-month nontreatment periods. Improvements in both the frequency of VM episodes and the interictal symptoms suggest a common underlying cause that was influenced by treatment including MCT.

Any attempt to explain why the clinical presentation of the participants in this study improved demands a cautious approach, not only because of the limitations inherent in case study design but also because the pathophysiology of VM remains uncertain ( Mallampalli *et al.*, 2021). It is this uncertainty that instigated a panel of experts to call for collaboration to elucidate the pathophysiological mechanisms underlying VM ( Mallampalli *et al.*, 2021) and provide the conditions for which case study research is well suited. That is, to contribute to the broader scientific discussion around the potential pathophysiology of a disease, particularly when the view may be seen as counterintuitive or contrarian ( Vandenbroucke, 2001). Speculation regarding the possible reason for effects observed with our three participants serves to propose novel directions for future investigation.

The focus of our hypothesis centres around a key finding in all our participants: palpable asymmetries of the upper cervical spine and resistance to transverse pressure on the spinous process of C2. In combination, these findings were clinically interpreted to represent hypertonicity of the ipsilateral obliquus capitis inferior (OCI) (a hypothesis requiring validation). Of the many CNS regions that are potentially affected by this purported muscle dysfunction, it is interesting to note that the locus coeruleus (LC) has been shown to be sensitive to small asymmetries in trigeminal muscle afferent input ( De Cicco *et al.*, 2014), the correction of which has a demonstrable impact on cognitive function ( De Cicco *et al.*, 2016). Beyond cognition, the LC has been linked to numerous aspects of migraine pathophysiology, including cortical spreading depression (purported to underpin migraine aura ( Goadsby *et al.*, 2017)) and pain modulation ( Vila-Pueyo *et al.*, 2019), plays a role in anxiety and depression ( Poe *et al.*, 2020), and has been linked to the comorbidity of anxiety with vestibular disorders ( Balaban *et al.*, 2011). In this light, it is interesting to note that the asymmetries and resistance noted at C2 were resolved during treatment with sustained pressures on the spinous process of C3 and coincided with changes in our participants’ primary and/or secondary outcome measures, providing a direction for further research.

While it is clear that the participants in this case series improved (see Table 1– Table 3 and Supplementary file 1), it is impossible to attribute improvement to a specific aspect of treatment. The ‘real world’ nature of these treatments encompass a multimodal approach, where MCT is provided in conjunction with education and a single stretching exercise. It is therefore possible that any one of these modalities, some of them in concert, or indeed none of them (placebo or natural history) rather than MCT alone, is responsible for the changes observed. Indeed, participant 1 was using migraine prophylaxis (venlafaxine and propranolol) and had these dosages increased in the follow-up period A, after a significant reduction in symptoms had already occurred during period B.

As a case series, we are limited by the lack of a control group and sample size power and are open to the potential confounding factors associated with selection bias ( Nissen & Wynn, 2014). We have attempted to minimise bias by recruiting participants consecutively and prospectively, conducting this study in accordance with the CARE guidelines for reporting case studies and the CENT guidelines for reporting on N-of-1 trials ( Gagnier *et al.*, 2013; Vohra *et al.*, 2015). Previously, the natural history of VM was reported in an observational study of 61 patients followed up over nine years. Recurrent vertigo was observed in 87% of patients with 44% unchanged or worse and with 56% with some reduction in frequency ( Radtke *et al.*, 2012). While not impossible, spontaneous remission in three participants with long histories of symptoms seems highly implausible. Expectation of a positive outcome is crucial to a placebo response ( Benedetti, 2013). While it is well accepted that manual therapy (MT) provides a placebo effect ( Bialosky *et al.*, 2017), these effects have been assessed in conditions that would traditionally be treated with MT. As none of our participants experienced neck pain or restricted ROM supporting a ‘cervicogenic dizziness’ diagnosis and MCT is not considered a treatment for VM ( Byun *et al.*, 2021; Mallampalli *et al.*, 2021), it seems unlikely that these individuals had a ‘strong’ if any expectation of a positive result. The exception is participant P2, who had undergone chiropractic MT for many years for lower back problems. This included treatment of the neck, which provided relief for one to two days, possibly giving some belief that the neck was implicated.

## Conclusion

This case series demonstrates an observable, rapid and sustained impact on the clinical presentation and medium to large effects in self-reported symptoms of VM in three prospectively and consecutively recruited patients. VM is a relatively new diagnostic category with currently unclear pathophysiology and treatment pathways. It is therefore important that all hypotheses regarding pathophysiology be considered and tested and that novel treatment options be considered. Despite the known pathoanatomical and pathophysiological pathways linking the upper cervical spine to regions explaining symptom behaviour, the upper cervical spine is not readily considered in current pathophysiological explanations for VM. The challenge of proposing a hypothesis involving the upper cervical spine for conditions involving migraine and vertigo is not lost on the authors. In light of this, we echo the sentiments of May ( May, 2020), urging readers to remain open to all possible mechanisms associated with migraine and vertigo to inform a broader approach to managing these patients.

In conclusion, this case series suggests that this MCT approach may represent a safe, well-tolerated and affordable option with the potential for rapid and sustained relief of symptoms in some patients with VM and advocates a legitimate avenue of scientific enquiry for treatment and pathophysiological mechanisms of VM.

## Data Availability

No data are associated with this article. Open Science Framework: Manual Cervical Therapy and Vestibular Migraine, https://doi.org/10.17605/OSF.IO/23MNJ ( O'Toole, 2023). This project contains the following underlying data: Supplementary file 1 Manual Cervical Therapy and Vestibular Migraine ( *Clinical outcome measures – tabulated symptom diary data and patient reported outcome measure data for 3 participants from baseline to 6 months post treatment, recorded in excel spreadsheet to calculate Cohen's D and Hedges' g*) Participant Statement – P01 (Statement from study participant 01 regarding impact of disease, management prior to and during the study period) Participant Statement – P02 (Statement from study participant 02 regarding impact of disease, management prior to and during the study period) Data are available under the terms of the Creative Commons Zero "No rights reserved" data waiver (CC0 1.0 Public domain dedication).
