# Supplementary material for: Manual cervical therapy and vestibular migraine: A case series
Source: Health Open Res. 2023 Aug 22;5:12. Originally published 2023 May 22. [Version 3] doi: 10.12688/healthopenres.13319.3 (PMC11065132; doi:10.12688/healthopenres.13319.3)
Supplement: Peer review history

# Reviewer report: Reviewer response for version 3

**DOI:** 10.21956/healthopenres.14501.r27457

**Published:** 2023-10-16

**Authors:** Toby M. Hall

**Affiliations:** 1 Curtin School of Allied Health, Curtin University, Perth, Western Australia, Australia

The authors have address all my concerns and I think those of the previous reviewers.

Are enough details provided of any physical examination and diagnostic tests, treatment given and outcomes?

Partly

Is the background of the cases’ history and progression described in sufficient detail?

Yes

Is sufficient discussion included of the importance of the findings and their relevance to future understanding of disease processes, diagnosis or treatment?

Yes

Is the conclusion balanced and justified on the basis of the findings?

Yes

Reviewer Expertise:

Manual examination. Diagnostic accuracy. Manual therapy. Cervicogenic headache

I confirm that I have read this submission and believe that I have an appropriate level of expertise to confirm that it is of an acceptable scientific standard.

# Reviewer report: Reviewer response for version 3

**DOI:** 10.21956/healthopenres.14501.r27456

**Published:** 2023-08-24

**Authors:** Julia Treleaven

**Affiliations:** 1 Neck and Head Research Unit, The School of Health and Rehabilitation Sciences, The University of Queensland, Saint Lucia, Queensland, Australia

I have now reviewed and approve the revised version.

Are enough details provided of any physical examination and diagnostic tests, treatment given and outcomes?

Partly

Is the background of the cases’ history and progression described in sufficient detail?

Partly

Is sufficient discussion included of the importance of the findings and their relevance to future understanding of disease processes, diagnosis or treatment?

Partly

Is the conclusion balanced and justified on the basis of the findings?

Partly

Reviewer Expertise:

NA

I confirm that I have read this submission and believe that I have an appropriate level of expertise to confirm that it is of an acceptable scientific standard.

# Reviewer report: Reviewer response for version 2

**DOI:** 10.21956/healthopenres.14448.r27404

**Published:** 2023-08-07

**Authors:** Toby M. Hall

**Affiliations:** 1 Curtin School of Allied Health, Curtin University, Perth, Western Australia, Australia

This is an interesting and generally well written paper which has improved in its second version. The authors have responded to the reviewer’s comments in the main. There is now some modification to the introduction with an expanded section on current treatment for vestibular migraine and a discussion on manual therapy for migraine.

 Assessment information for each participant has been included more comprehensively, although I would like to see more information on the test used to identify suitable patients for this treatment. That is the transverse pressure of the C2 spinous process. Mainly in the interests of future research on this approach. What is the reliability of this test? What is actually an "asymmetrical response". Is it pain and stiffness? Or both?

 The discussion now highlights the resolution of symptoms with upper cervical intervention and attempts to relate this to existing literature relevant to sub-occipital muscles. However I feel that more detailed explanation needs to be given on how pressure to C3 spinous process alone might alter sub-occipital muscle activation. The authors quote Watson & Drummond (2014) to support the application of pressure to C3 but in that paper pressure was not just applied to C3. Pain or Vestibular migraine is clearly a complex issue. Can this complexity really be explained by changes to these small muscles alone by the application of force to a single vertebra? Its important to be as clear as possible in the explanation of potential effects. More evidence is clearly required and as the authors point out, very little can be taken from a case series other than to inform on future research.  

 I also have some concerns, in line with the first reviewer’s comment, about the recognition of potential bias of the participants in their very likely positive expectation for a positive outcome from treatment they expected to receive attending a specialist clinic.

Are enough details provided of any physical examination and diagnostic tests, treatment given and outcomes?

Partly

Is the background of the cases’ history and progression described in sufficient detail?

Yes

Is sufficient discussion included of the importance of the findings and their relevance to future understanding of disease processes, diagnosis or treatment?

Yes

Is the conclusion balanced and justified on the basis of the findings?

Yes

Reviewer Expertise:

Manual examination. Diagnostic accuracy. Manual therapy. Cervicogenic headache

I confirm that I have read this submission and believe that I have an appropriate level of expertise to confirm that it is of an acceptable scientific standard, however I have significant reservations, as outlined above.

# Response

**Published:** 2023-08-14

**Authors:** Roger O'Toole

**Affiliations:** Melbourne Headache Centre, Melbourne, Victoria, Australia

This is an interesting and generally well written paper which has improved in its second version. The authors have responded to the reviewer’s comments in the main. There is now some modification to the introduction with an expanded section on current treatment for vestibular migraine and a discussion on manual therapy for migraine. **Thank you for your time. We greatly appreciate the time taken to review this article and your feedback to improve its quality.**  

 Assessment information for each participant has been included more comprehensively, although I would like to see more information on the test used to identify suitable patients for this treatment. **Thank you for your clarifying question. Participants were selected prior to manual (or any other) clinical assessment based on their diagnosis and absence of exclusion criteria as indicated in Eligibility criteria.**  

 That is the transverse pressure of the C2 spinous process. Mainly in the interests of future research on this approach. What is the reliability of this test? What is actually an "asymmetrical response". Is it pain and stiffness? Or both? **To the best of our knowledge transverse pressure has not been tested as an assessment tool. The palpation of bony position of C2 spinous process and resistance to transverse pressure from left, compared to right have not been formally tested for reliability and validated as relating to OCI hypertonicity. Whilst the lack of validation was mentioned in the discussion the absence of formally tested reliability was not and is an omission that has been corrected. The discussion now reflects this as an area of interest for the next stages of research.** **Asymmetry is described only in terms of resistance to transverse pressure. Pain on palpation is not described and not necessary for assessment, as the aim is to determine the side of OCI hypertonicity.**  

 The discussion now highlights the resolution of symptoms with upper cervical intervention and attempts to relate this to existing literature relevant to sub-occipital muscles. However I feel that more detailed explanation needs to be given on how pressure to C3 spinous process alone might alter sub-occipital muscle activation. **Thank you for sharing reviewer 1’s interest in our hypothesis regarding the possible mechanism by which C2-3 may cause, and be implicated in the treatment of dysfunction relating to OCI. We have expanded the discussion to further highlight our hypothesis in this regard.**   

 The authors quote Watson & Drummond (2014) to support the application of pressure to C3 but in that paper pressure was not just applied to C3. **Thank you for seeking clarification. The introduction has been modified to remove reference to the techniques described by Watson and Drummond (2014) which aimed to reproduce familiar headpain. The last paragraph now reflects the description under interventions for the technique for central PA pressure on C3 is cited in both sections as Watson, 1997 referring to the training manual for learning the technique. This is the first published description of that aspect of the treatment approach.**

 Pain or Vestibular migraine is clearly a complex issue. Can this complexity really be explained by changes to these small muscles alone by the application of force to a single vertebra? Its important to be as clear as possible in the explanation of potential effects. More evidence is clearly required and as the authors point out, very little can be taken from a case series other than to inform on future research. **Thank you for your question. Firstly, pain and migraine in any manifestation is a complex issue, and this is reflected in the incomplete resolution of symptoms in all three participants. This is not being presented as a ‘simple neck issue’, but moreso an important and overlooked cog in a complex wheel.  Secondly, we have yet to prove that these changes occurred due to the application of pressure to C3, as this was a multimodal therapy. I have no doubt, as reviewer 2 has indicated, that education and a single stretch played a role in achieving the response we have reported, but we also have acknowledged potential confounders with natural history and placebo.    Your disbelief at the potential impact of a minimalist intervention is mirrored by and exceeded on a daily basis by the people we treat. The introduction and discussion adequately highlight the possible networks disturbed by dysfunction affecting OCI connecting it to all possible symptomatic manifestations of VM. The aim of a case series is to raise questions rather than provide answers, and I am excited at the interest that three well credentialled reviewers have shown in this paper and look forward to sharing the next steps of the research journey to demonstrate how impactful small changes in the sub-occipital region can be.**  

 I also have some concerns, in line with the first reviewer’s comment, about the recognition of potential bias of the participants in their very likely positive expectation for a positive outcome from treatment they expected to receive attending a specialist clinic. **Thank you for sharing reviewer 1’s input. We agree that there is certainly a selection bias impacting generalisability of results and this will also reflect an underlying belief the treatment could be of benefit. The last paragraph has been amended to reflect this.**

# Reviewer report: Reviewer response for version 2

**DOI:** 10.21956/healthopenres.14448.r27401

**Published:** 2023-07-20

**Authors:** Kerstin Luedtke

**Affiliations:** 1 Department of Orthopedics and Trauma Surgery, Academic Physiotherapy, Pain and Exercise Research, University of Luebeck, Luebeck, Germany

Thank you very much for reporting these three interesting cases of vestibular migraine. All three have complex clinical presentations combining long disease histories and co-morbidities with traumatic events and psychiatric diagnoses. All three cases improved after sustained manual techniques.

 The manuscript is well written and provides sufficient detail about patient cases and interventions. The discussion is appropriately worded for a case series. The authors acknowledge that 3 cases cannot provide evidence for the effect of the intervention and that future research is required to investigate whether manual therapy can effectively reduce vestibular migraine symptoms.

 I have only one comment which I believe should find a place in the discussion section: the treatment did not only provide sustained pressure on C3 but also education about the disease and a self-management program. Both are extremely relevant treatment components. We just showed, that simply explaining the pathophysiology of migraine when provided additionally to manual therapy enhanced the effectiveness of the treatment (Meise et al. 2023) ^1^. And from other chronic pain diseases we know, that regaining control over symptoms by being provided with a home exercise program contributes to symptom and disability reduction. I would love to see this discussed more in depth.

Are enough details provided of any physical examination and diagnostic tests, treatment given and outcomes?

Partly

Is the background of the cases’ history and progression described in sufficient detail?

Yes

Is sufficient discussion included of the importance of the findings and their relevance to future understanding of disease processes, diagnosis or treatment?

Yes

Is the conclusion balanced and justified on the basis of the findings?

Yes

Reviewer Expertise:

Physiotherapy, pain, migraine

I confirm that I have read this submission and believe that I have an appropriate level of expertise to confirm that it is of an acceptable scientific standard, however I have significant reservations, as outlined above.

## References

[1] : Additional effects of pain neuroscience education combined with physiotherapy on the headache frequency of adult patients with migraine: A randomized controlled trial. *Cephalalgia* .2023;43(2) : 10.1177/03331024221144781 3331024221144781 10.1177/03331024221144781 36739510

# Response

**Published:** 2023-08-14

**Authors:** Roger O'Toole

**Affiliations:** Melbourne Headache Centre, Melbourne, Victoria, Australia

Thank you very much for reporting these three interesting cases of vestibular migraine. All three have complex clinical presentations combining long disease histories and co-morbidities with traumatic events and psychiatric diagnoses. All three cases improved after sustained manual techniques. The manuscript is well written and provides sufficient detail about patient cases and interventions. The discussion is appropriately worded for a case series. The authors acknowledge that 3 cases cannot provide evidence for the effect of the intervention and that future research is required to investigate whether manual therapy can effectively reduce vestibular migraine symptoms. Thank you for your time. We greatly appreciate the time taken to review this article and your feedback to improve its quality.   I have only one comment which I believe should find a place in the discussion section: the treatment did not only provide sustained pressure on C3 but also education about the disease and a self-management program. Both are extremely relevant treatment components. We just showed, that simply explaining the pathophysiology of migraine when provided additionally to manual therapy enhanced the effectiveness of the treatment (Meise et al. 2023) 1. And from other chronic pain diseases we know, that regaining control over symptoms by being provided with a home exercise program contributes to symptom and disability reduction. I would love to see this discussed more in depth. Thank you for seeking clarification regarding the multimodal nature of the intervention, and providing further resources to support this. We had acknowledged that as a real-world intervention it was multi-modal with education and a single exercise complementing the manual therapy. We have expanded and reworded the discussion to incorporate your finding, that education can enhance the effectiveness of manual therapy and to emphasise that it is more likely a combination of modalities (MT + education + stretch) than manual therapy alone that has had a beneficial impact.

# Reviewer report: Reviewer response for version 2

**DOI:** 10.21956/healthopenres.14448.r27386

**Published:** 2023-07-19

**Authors:** Julia Treleaven

**Affiliations:** 1 Neck and Head Research Unit, The School of Health and Rehabilitation Sciences, The University of Queensland, Saint Lucia, Queensland, Australia

I am happy with most of the corrections. I have a couple of comments that should be addressed before approval.

1. Something should be mentioned about how the treatment of C3 pressure might effect the IO muscle given its attachments. The discussion centres around the fact that I/O muscle is likely implicated but the potential  effect of the treatment should be mentioned.
2. Bias of the participants- it should still be stated that the participants were attending and paying for private physiotherapy so presumably they had some belief the treatment might work.

Both of these should be addressed in the next version.

Are enough details provided of any physical examination and diagnostic tests, treatment given and outcomes?

Partly

Is the background of the cases’ history and progression described in sufficient detail?

Partly

Is sufficient discussion included of the importance of the findings and their relevance to future understanding of disease processes, diagnosis or treatment?

Partly

Is the conclusion balanced and justified on the basis of the findings?

Partly

Reviewer Expertise:

NA

I confirm that I have read this submission and believe that I have an appropriate level of expertise to confirm that it is of an acceptable scientific standard, however I have significant reservations, as outlined above.

# Reviewer report: Reviewer response for version 1

**DOI:** 10.21956/healthopenres.14403.r27221

**Published:** 2023-06-09

**Authors:** Julia Treleaven

**Affiliations:** 1 Neck and Head Research Unit, The School of Health and Rehabilitation Sciences, The University of Queensland, Saint Lucia, Queensland, Australia

**Major comments**

 This is generally an interesting paper. There are major revisions required to improve the validity of this paper (see below). 

**SPECIFIC COMMENTS**

**INTRODUCTION -**

 Overall, the introduction contains a lot of information that is likely irrelevant to the topic and certainly doesn’t match the possible hypothesis of the discussion. I suggest that it is rewritten to reflect this.  

 Second paragraph - co-morbidity doesn’t equal causality. Might be better to rephrase to suggest that only many migraine patients suffer from dizziness that might also be a spectrum of VM and thus the incidence could be higher.

 Third paragraph need to first establish the diagnostic criteria of VM and then show the similarity to whiplash. Cervical vertigo is not a suitable term, only 25 % of the whiplash patients reported vertigo.

 Fourth paragraph - greater MSK is not supported by a recent SR- Liang et al 2019. (Liang, Galea et al. 2019) ^1^. This sentence is not needed to make the point from previous paragraph supporting cervical afferents for contributing to the cause of dizziness. Suggest remove the first sentence.

 Could also suggest a possible trigger for migraine, rather than a direct cause of the symptoms e.g. (Malhotra, Pace et al. 2020) ^2^.   Could also refer to recent articles suggesting a possible autonomic trigger from the upper cervical region. (Hammerle, Lu et al. 2022, Hammerle, Thomas et al. 2022) ^3^^,^^4^.

 Paragraph 7 – need to discuss current treatment evidence for VM (Byun, Levy et al. 2021) ^5^ as well as current evidence for manual therapy for usual migraine as well.  There have been some trials e.g. (Chaibi, Tuchin et al. 2011, Herranz-Gómez, García-Pascual et al. 2021, Varangot-Reille, Suso-Martí et al. 2022) ^6^^,^^7^^,^^8^.  Thus the aim of the study is to demonstrate effect of this specific MCT in VM.  Need to also discuss this particular technique and approach and how it is different. 

**METHODS -**

 Ethical clearance required?

 This sentence does not make sense – please clarify. “Subsequently, absence of neck pain and/or limitation of cervical ROM in all three participants eliminated concurrent ‘cervicogenic dizziness’ as an alternate or comorbid diagnosis (Reiley et al., 2017).”

 Need reference for 50% reduction in frequency.

 Is the hit 6 most suitable for VM- headache? 

 Need more information re: the diary. How collated etc.

 Intervention- should state all that was done here included stretches etc.

 The manual technique is a form of MT and should also be introduced in the introduction.

 Not clear what the difference between migraine, headaches, internal and external vertigo. These measures need to be described in the methods.

**RESULTS -**

 P1- was there a history of normal migraine prior?

 Table 1 – need to be clear about what all these are e.g. is headache number of headaches in the 28 days?  Also ensure all relating to clinically meaningful change is depicted in the tables as well. Interventions state it was PA pressure at C3 but in patient 1 it was transverse pressure to C2- or is that just assessment findings? 

 Also dose of propranolol significantly increased during that time as well. Why was this done? This should be mentioned in the results.

 P2. Why was sustained neck rotation done? Was this also done in P1?  Reference this test?

 P3 had a hit to the back of the head would that be exclusion? (Participants were excluded from the study if they had a history of previous neck surgery or traumatic head injury”.  Perhaps state why this was an exclusion criteria in the first place or refine the statement?

 Tables should be uniform. Perhaps put primary outcome measures first and then secondary outcome measures.

 Present summary of effect sizes as well in results.

**DISCUSSION -**

 Discussion is better description of possible relationships between the neck and VM in these patients than the introduction. This further highlights that a lot of the introduction is not necessary as it doesn’t relate to the discussion.  Could also refer to sustained cervical rotation and these articles (Hammerle, Lu et al. 2022, Hammerle, Thomas et al. 2022) to help your point in the introduction and revisit in the discussion ^3^^,^^4^.

 Need to sate why Patient 1 increased dose of medication.

 ”it seems unlikely that these individuals had a ‘strong’ if any expectation of a positive result.”-  but how were the patents referred to the clinic? They sought and paid for treatment so there was likely some belief that it would work.

 Perhaps stress in the conclusion it might only be some patients, some bias in selection of VM patients.

Are enough details provided of any physical examination and diagnostic tests, treatment given and outcomes?

Partly

Is the background of the cases’ history and progression described in sufficient detail?

Partly

Is sufficient discussion included of the importance of the findings and their relevance to future understanding of disease processes, diagnosis or treatment?

Partly

Is the conclusion balanced and justified on the basis of the findings?

Partly

Reviewer Expertise:

neck pain, dizziness, headache, concussion. I am not an expert at n of 1 trials.

I confirm that I have read this submission and believe that I have an appropriate level of expertise to confirm that it is of an acceptable scientific standard, however I have significant reservations, as outlined above.

## References

[1] : Cervical musculoskeletal impairments in migraine and tension type headache: A systematic review and meta-analysis. *Musculoskelet Sci Pract* .2019;42: 10.1016/j.msksp.2019.04.007 67-83 10.1016/j.msksp.2019.04.007 31054485

[2] : Headaches in hypermobility syndromes: A pain in the neck?. *Am J Med Genet A* .2020;182(12) : 10.1002/ajmg.a.61873 2902-2908 10.1002/ajmg.a.61873 32940405

[3] : Possible autonomic or cranial nerve symptoms triggered during sustained neck rotation in persistent headache post-concussion: a retrospective observational cross-sectional study. *J Man Manip Ther* .2023;31(2) : 10.1080/10669817.2022.2085850 113-123 10.1080/10669817.2022.2085850 35695356 PMC10013563

[4] : Sub-occipital muscle pressure pain thresholds correlate to direction of symptomatic active comfortable sustained neck rotation testing in post-concussive headache: a retrospective observational cross-sectional study. *J Man Manip Ther* .2023;31(2) : 10.1080/10669817.2022.2122370 124-129 10.1080/10669817.2022.2122370 36102346 PMC10013433

[5] : Treatment of Vestibular Migraine: A Systematic Review and Meta-analysis. *Laryngoscope* .2021;131(1) : 10.1002/lary.28546 186-194 10.1002/lary.28546 32083732

[6] : Manual therapies for migraine: a systematic review. *J Headache Pain* .2011;12(2) : 10.1007/s10194-011-0296-6 127-33 10.1007/s10194-011-0296-6 21298314 PMC3072494

[7] : Effectiveness of Exercise and Manual Therapy as Treatment for Patients with Migraine, Tension-Type Headache or Cervicogenic Headache: An Umbrella and Mapping Review with Meta-Meta-Analysis. *Applied Sciences* .2021;11(15) : 10.3390/app11156856 10.3390/app11156856

[8] : Exercise and Manual Therapy for the Treatment of Primary Headache: An Umbrella and Mapping Review. *Phys Ther* .2022;102(3) : 10.1093/ptj/pzab308 10.1093/ptj/pzab308 35084039

# Response

**Published:** 2023-06-22

**Authors:** Roger O'Toole

**Affiliations:** Melbourne Headache Centre, Melbourne, Victoria, Australia

Major comments

 This is generally an interesting paper. There are major revisions required to improve the validity of this paper (see below). **Thank you for your positive feedback. As a clinical therapist venturing into research I greatly appreciate the amount of time you put into feedback. Your contribution has (we hope) improved the scientific validity of this paper.**

 SPECIFIC COMMENTS

 INTRODUCTION -

 Overall, the introduction contains a lot of information that is likely irrelevant to the topic and certainly doesn’t match the possible hypothesis of the discussion. I suggest that it is rewritten to reflect this. 

 Second paragraph - co-morbidity doesn’t equal causality. Might be better to rephrase to suggest that only many migraine patients suffer from dizziness that might also be a spectrum of VM and thus the incidence could be higher. **Thank you for your comment. As a symptom, dizziness is almost entirely excluded (other than when associated with head movement and nausea) from the classification of VM. The reporting of the co-morbidity between dizziness and migraine was not intended to suggest causality, but more to extend the understanding of the relationship beyond the reported prevalence of VM. While I agree these probably represent a spectrum, I also agree that it doesn’t add to the hypothesis of this paper and have duly been omitted.**

 Third paragraph need to first establish the diagnostic criteria of VM and then show the similarity to whiplash. Cervical vertigo is not a suitable term, only 25 % of the whiplash patients reported vertigo. **Thank you for your feedback. I can see the confusion in my wording indicating a more direct comparison of VM to WAD which was not the intent.The paragraph was intended to highlight a discordant view that despite clinical and experimental evidence, the upper cervical spine is not considered capable of (or rarely) causes the symptom of vertigo, and subsequently would not contribute to VM as a disorder. I have edited the paragraph to refocus the content on vertigo and upper cervical afferents. I understand there are nosological debates around suitable naming of different disorders. I am comfortable citing the term ‘cervical vertigo’ as this was the label the authors used in their paper proposing a new disorder, although I acknowledge this is a source of contention.**  

 Fourth paragraph - greater MSK is not supported by a recent SR- Liang et al 2019. (Liang, Galea et al. 2019) 1. This sentence is not needed to make the point from previous paragraph supporting cervical afferents for contributing to the cause of dizziness. Suggest remove the first sentence.

 Could also suggest a possible trigger for migraine, rather than a direct cause of the symptoms e.g. (Malhotra, Pace et al. 2020) 2.   Could also refer to recent articles suggesting a possible autonomic trigger from the upper cervical region. (Hammerle, Lu et al. 2022, Hammerle, Thomas et al. 2022) 3, 4. **Thank you for highlighting the current lack of evidence establishing greater msk dysfunction in migraine. The opening sentence has been removed.**

 Paragraph 7 – need to discuss current treatment evidence for VM (Byun, Levy et al. 2021) 5 as well as current evidence for manual therapy for usual migraine as well.  There have been some trials e.g. (Chaibi, Tuchin et al. 2011, Herranz-Gómez, García-Pascual et al. 2021, Varangot-Reille, Suso-Martí et al. 2022) 6, 7, 8.  Thus the aim of the study is to demonstrate effect of this specific MCT in VM.  Need to also discuss this particular technique and approach and how it is different. **Thank you for your suggestion. We have expanded the current treatment evidence for VM and included a review of manual therapy for migraine. We have also included a description of the manual therapy used in this study.**

 METHODS -

 Ethical clearance required?

 This sentence does not make sense – please clarify. “Subsequently, absence of neck pain and/or limitation of cervical ROM in all three participants eliminated concurrent ‘cervicogenic dizziness’ as an alternate or comorbid diagnosis (Reiley et al., 2017).” **Thank you for your feedback. We have revised the sentence for clarity.**

 Need reference for 50% reduction in frequency. **Thank you for pointing out this omission. A reference to the guidelines for reporting clinical trials has been added.**

 Is the hit 6 most suitable for VM- headache? **Thank you for your question. All participants had significant headache interictally, with headache recorded 20, 17 and 23 days per 28 days respectively. This justifies the use of a headache specific PRO and the HIT-6 is a valid and reliable tool for assessing the impact of headache symptoms. However, with each patient exhibiting multiple interictal symptoms it is likely that the specificity of attribution of impact to headache may not be valid. This is highlighted by Participant 1’s HIT-6 increasing to ‘severe impact’ in the 4 weeks from 2-3 months post treatment, despite only recording 1 headache for the period with the same intensity as the previous period (NRS 2/10). That other life events impacted during this period is reported, as reflected in the DASS-21 indicating the headache, despite being less frequent and just as intense, was more impactful. Future research will incorporate more objective measures to compliment clinical outcomes and PRO’s.**  

 Need more information re: the diary. How collated etc. **Thank you for highlighting this. We have added further detail on the collating and confirmation of diary data in the methods section.**   I

 ntervention- should state all that was done here included stretches etc. **Thank you for your suggestion. We have added further detail in the Interventions section regarding the stretch and education. ** 

 Not clear what the difference between migraine, headaches, internal and external vertigo. These measures need to be described in the methods. **Thank you for your question. We have added further detail on the definition of each symptom in the methods section.**

 The manual technique is a form of MT and should also be introduced in the introduction. **Thank you for your suggestion. We have added further detail in the introduction and methods on the technique used.**

 RESULTS -

 P1- was there a history of normal migraine prior? **Thank you for your question. No, there was no past history of migraine in participant 1.**

 Table 1 – need to be clear about what all these are e.g. is headache number of headaches in the 28 days?  Also ensure all relating to clinically meaningful change is depicted in the tables as well. **Thank you for your observation. The formatting of the tables with alternating highlights may be confusing and the headings not standing out as well as desired.  We have requested an amenedment to better emphasise the heading for each section.**

 Interventions state it was PA pressure at C3 but in patient 1 it was transverse pressure to C2- or is that just assessment findings? **A key part of the assessment is bony position of the C2 spinous process and asymmetrical resistance with transverse pressure at C2. Clinically this is interpreted as increased tone of OCI unilaterally and is explained in paragraph 3 of the discussion. The intervention to treat or correct this is a PA pressure on the spinous process of C3. An explanation of the assessment of C2 will be added to the introduction and methods section describing the technique.**

 Also dose of propranolol significantly increased during that time as well. Why was this done? This should be mentioned in the results. **Thank you for this observation, as not commenting on this is an oversight. Participant 1 suffered from comorbid anxiety and depression (omitted), which were exacerbated with events surrounding COVID lockdown. Originally prescribed propranolol and effexor for her VM, when she resumed taking them seven months prior to assessment (described in the results under P1), they did not help her VM symptoms, but were helping manage her anxiety.  She was self titrating effexor beads (hence the variable dose) and increased prorpanolol when her anxiety increased. As the table of results indicates significant change across all outcomes measures except for nausea before the increase in dose of either medication we did not expand on the explanation, but in reflection this was an oversight that will be revised.**

 P2. Why was sustained neck rotation done? Was this also done in P1?  Reference this test? **For clarification we have amneded the assessment section for each participant, as some data realting to testing in all 3 was omitted. This represents part of VBI screening. Data regarding findings for this test have been added to the results for each participant.**

 P3 had a hit to the back of the head would that be exclusion? (Participants were excluded from the study if they had a history of previous neck surgery or traumatic head injury”.  Perhaps state why this was an exclusion criteria in the first place or refine the statement? **Thank you for your observation. The error is with the wording of the exclusion criteria which should read traumatic brain injury. P3's head hit a branch whilst straightening from leaning under it. It was offerred as an event based on a momentary feeling of 'strain' in the neck P3 though tmight be relevant to the neck assessment, rather than an impact to the head. There were no symptoms beyond the initial neck strain feeling, relating either to the head or neck. In a short time after he had a viral illness. On re-reading the temporal relationship of this episode is not as clear as it could be and we will revise the description for P3, but are confident that the incident did not reflect a concussion/mTBI and therefore warrant exclusion.**  

 Tables should be uniform. Perhaps put primary outcome measures first and then secondary outcome measures. **Thank you for your comments on the table. Being a case sewries we tried to reflect the variability amonst patient symptom intensity and frequency, and medication use as uniformly as possible. These are listed as our primary outcome measures with the PRO's as secondary outcomes, listed at the bottom of each table.**

 Present summary of effect sizes as well in results. **Access to the full data set is available under Extended data at the end of the manuscript.**

 DISCUSSION -

 Discussion is better description of possible relationships between the neck and VM in these patients than the introduction. This further highlights that a lot of the introduction is not necessary as it doesn’t relate to the discussion.  Could also refer to sustained cervical rotation and these articles (Hammerle, Lu et al. 2022, Hammerle, Thomas et al. 2022) to help your point in the introduction and revisit in the discussion 3, 4. **Thank you for highlighting this recent evidence. We have included them in the expanded discussion on the response of symptoms in sustained cervical rotation to treatment.**

 Need to sate why Patient 1 increased dose of medication. **See prev comments.**  

 ”it seems unlikely that these individuals had a ‘strong’ if any expectation of a positive result.”-  but how were the patents referred to the clinic? They sought and paid for treatment so there was likely some belief that it would work. **I can understand how this might seem incongruous, and ultimately the question of placebo will require suitable trial conditions to prove or disprove, rather than our interpretation of expectations. That said, the current medical model says that vertigo is not a symptom produced by the upper cervical spine, and that migraine is a neurological disorder best treated by neurologists and doctors with medications. We see people in whom these approaches have failed, often multiple times over many years. My own opinion and clinical experience is that ‘high hopes’ the treatment will help is a long way from ‘strong expectation of positive benefit’. P2 is a great example (refer to her client impact statement). Decades of misdiagnosis and mistreatment built a significant distrust of the medical system, and previous manual therapy failure to improve her symptoms may in fact argue for a ‘nocebo’ effect. As indicated she may have had some expectation of positive benefit, but equally, we could make a case for her having an expectation of short term benefit as that had been her experience with manual therapy prior. Equally P1 could have had strong beleifs that effexor (venlafaxine) would help, as it had done so previously, yet it failed to improve her symptoms. A more robust design is required to elucidate the relative contribution of placebo, manual therapy, education and natural history to any changes observed. Our puropse was simply to have upper cervical manual assessmen included in that disucssion.**

 Perhaps stress in the conclusion it might only be some patients, some bias in selection of VM patients. **Yes a cautious tone has been foremost in our thoughts given the position Case Series occupy in the hierarchy of evidence. The final paragraph in the discussion acknowledges possible counfounding factors associated with selection bias, natural history, and placebo. The final paragraph of the conclusion does state 'in some patients with VM' which we believe adequately understate our findings.**

# Response

**Published:** 2023-08-14

**Authors:** Roger O'Toole

**Affiliations:** Melbourne Headache Centre, Melbourne, Victoria, Australia

1. Something should be mentioned about how the treatment of C3 pressure might effect the IO muscle given its attachments. The discussion centres around the fact that I/O muscle is likely implicated but the potential  effect of the treatment should be mentioned.

**Thank you for your interest in our hypothesis regarding the possible mechanism by which C2-3 may cause, and be implicated in the treatment of dysfunction relating to OCI. We have expanded the discussion to further highlight our hypothesis in this regard.  **

1. Bias of the participants- it should still be stated that the participants were attending and paying for private physiotherapy so presumably they had some belief the treatment might work.

**We agree that there is certainly a selection bias impacting generalisability of results and this will also reflect an underlying belief the treatment could be of benefit. The last paragraph has been amended to reflect this.**
